# Anticipating and blocking HIV-1 escape by second generation antiviral shRNAs

**DOI:** 10.1186/1742-4690-7-52

**Published:** 2010-06-08

**Authors:** Nick CT Schopman, Olivier ter Brake, Ben Berkhout

**Affiliations:** 1 Laboratory of Experimental Virology, Department of Medical Microbiology, Center for Infection and Immunity Amsterdam (CINIMA), Academic Medical Center, University of Amsterdam, Meibergdreef 15, 1105 AZ Amsterdam, The Netherlands

## Abstract

**Background:**

RNA interference (RNAi) is an evolutionary conserved gene silencing mechanism that mediates the sequence-specific breakdown of target mRNAs. RNAi can be used to inhibit HIV-1 replication by targeting the viral RNA genome. However, the error-prone replication machinery of HIV-1 can generate RNAi-resistant variants with specific mutations in the target sequence. For durable inhibition of HIV-1 replication the emergence of such escape viruses must be controlled. Here we present a strategy that anticipates HIV-1 escape by designing 2^nd ^generation short hairpin RNAs (shRNAs) that form a complete match with the viral escape sequences.

**Results:**

To block the two favorite viral escape routes observed when the HIV-1 integrase gene sequence is targeted, the original shRNA inhibitor was combined with two 2^nd ^generation shRNAs in a single lentiviral expression vector. We demonstrate in long-term viral challenge experiments that the two dominant viral escape routes were effectively blocked. Eventually, virus breakthrough did however occur, but HIV-1 evolution was skewed and forced to use new escape routes.

**Conclusion:**

These results demonstrate the power of the 2^nd ^generation RNAi concept. Popular viral escape routes are blocked by the 2^nd ^generation RNAi strategy. As a consequence viral evolution was skewed leading to new escape routes. These results are of importance for a deeper understanding of HIV-1 evolution under RNAi pressure.

## Background

Worldwide more than 30 million individuals are infected with human immunodeficiency virus type 1 (HIV-1) and each year approximately 3 million persons become newly infected. Treatment options have improved dramatically with the introduction of highly active antiretroviral therapy (HAART) that combines multiple antiviral drugs. However, long term HAART can have severe side effects, and the emergence of drug resistant viruses remains a possibility [1]. New durable antiviral strategies are needed, of which gene therapy based on RNA interference (RNAi) seems very promising. RNAi is an evolutionary conserved pathway in which double stranded RNA (dsRNA) mediates the sequence-specific degradation of a target RNA [2,3]. RNAi is triggered by small interfering RNA (siRNA), whereby the guide strand is incorporated into the RNA-induced silencing complex (RISC), while the passenger strand is degraded. The activated RISC complex directs the degradation of a fully complementary mRNA, resulting in silencing of the target gene [2,4-6].

RNAi can be used to inhibit virus replication by stable intracellular expression of anti-HIV short hairpin RNAs (shRNAs), which require processing into siRNAs by the Dicer endonuclease in the cytoplasm [7-14]. RNAi-based antiviral therapies have been developed and have entered clinical trials [15]. However, because the RNAi mechanism relies on sequence specificity, a virus with a high mutation rate such as HIV-1 is able to escape from the RNAi pressure by mutation of the target sequence [7,10,16,17]. For long-term suppression of HIV-1, the emergence of such escape variants must be controlled. Several strategies have been suggested to prevent viral escape, such as targeting of highly conserved and possibly immutable viral sequences, and the use of combinatorial RNAi approaches similar to HAART. Here we present an additional strategy to block favorite viral escape routes with 2^nd ^generation shRNAs that specifically recognize the mutated target sequences. This strategy requires up front knowledge of the viral escape options, which can than be anticipated by design of matching 2^nd ^generation shRNAs. We already demonstrated that HIV-1 escape is restricted when conserved genome sequences are targeted by RNAi [17]. In this study, we designed 2^nd ^generation shRNAs to block the two dominant escape routes observed when attacking HIV-1 sequences that encode the integrase enzyme. A combinatorial RNAi attack with three shRNAs against the wild type (wt) virus and the two escape variants was indeed able to restrict virus evolution.

## Results

### Design of 2^nd ^generation shRNAs that anticipate HIV-1 escape

**Figure 1:**
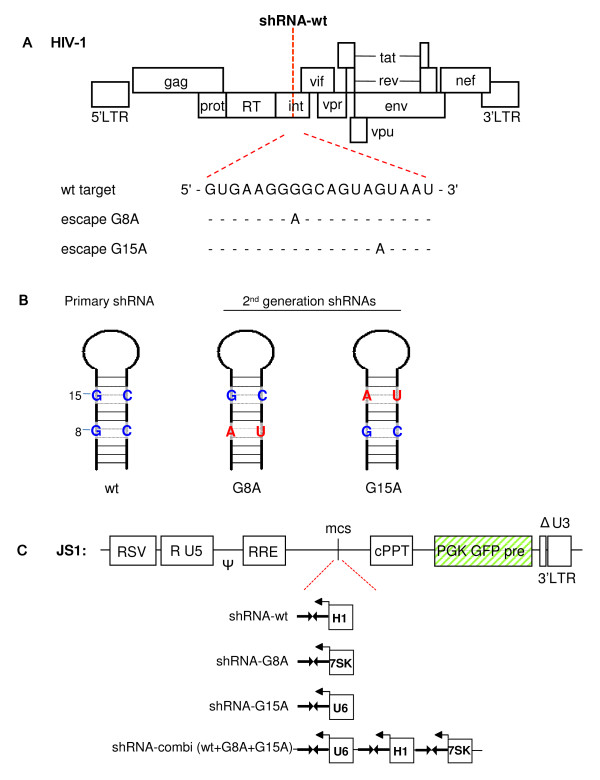
**Schematic of the HIV-1 genome and the shRNA inhibitors**. (A) The shRNA wt targets HIV-1 integrase (int). The wt target sequence is shown below together with the G8A and G15A escape mutations. (B) Depicted are the shRNAs against the integrase target. Indicated in red are the mutated nucleotides to construct the 2^nd ^generation shRNAs that target the G8A and G15A escape viruses. (C) The primary shRNA-wt and the 2^nd ^generation shRNA-G8A and shRNA-G15A cassettes were cloned in the lentiviral vector JS1 under control of the polymerase III promoters H1, 7SK and U6, respectively. All three shRNA cassettes were combined in the shRNA-combi vector.

In a previous study, we demonstrated that RNAi attack on conserved regions of the HIV-1 RNA genome allows the virus only a limited number of escape routes. In this study, we focused on the shRNA-wt inhibitor that targets sequences of the viral integrase gene, which previously yielded a severely restricted escape profile [17]. Two dominant escape routes were observed in massive virus evolution studies, and these escape variants have the G8A or G15A mutation in the target sequence (Fig.1A). We designed modified shRNAs that anticipate these two popular escape routes, the 2^nd ^generation shRNAs G8A and G15A (Fig. 1B). The gene cassettes encoding the primary shRNA-wt and the 2^nd ^generation inhibitors shRNA-G8A and shRNA-G15A were individually cloned in the lentiviral vector JS1 under control of the polymerase III promoters H1, 7SK and U6, respectively (Fig. 1C). In addition, all three shRNA cassettes were combined in the shRNA-combi vector. The use of different promoter elements is required to avoid recombination on repeat sequences during lentiviral transduction. We previously demonstrated equal shRNA expression levels from this vector using reporter assays and Northern blotting [18].

### Target knockdown by 2^nd ^generation shRNA is sequence-specific

**Figure 2:**
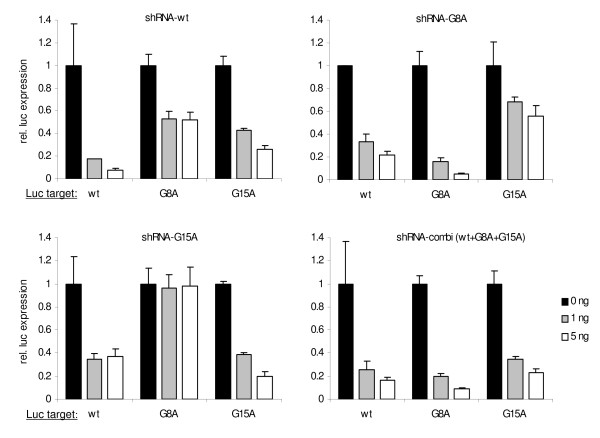
**Gene silencing by 2^nd ^generation shRNA is effective and sequence-specific**. (A) The effect of wt and 2^nd ^generation shRNA inhibitors on a luciferase reporter gene with the HIV-1 target sequence (wt, G8A or G15A). 293T cells were co-transfected with 25 ng firefly luciferase reporter plasmid (wt, G8A or G15A), 0.5 ng of renilla luciferase plasmid, and 0, 1 and 5 ng shRNA constructs. Relative luciferase activity were determined as the ration of the firefly and renilla luciferase expression. Values are shown as percentage of the transfection without shRNA. Averages and standard deviations represent at least three independent transfections that were performed in quadruple.

We first tested the activity and sequence specificity of the 2^nd ^generation shRNAs in co-transfection experiments in 293T cells with reporter constructs. We determined the inhibitory profile of the shRNAs (wt, G8A, G15A and combi) on three luciferase reporters (wt, G8A and G15A) with the HIV-1 integrase target sequence inserted in the 3'UTR. A renilla luciferase reporter plasmid was co-transfected to control for the transfection efficiency. The relative luciferase expression was determined as the ratio of the firefly and renilla luciferase activity. We transfected 2 amounts of the shRNA constructs (1 and 5 ng), and the luciferase values obtained without inhibitor were set at 1 for each construct (Fig. 2). The primary shRNA-wt caused a dramatic reduction of luciferase expression from the wt reporter, but significantly less reduction for the G8A and G15A reporters. Likewise, the 2^nd ^generation shRNAs inhibited the matching targets the best, thus demonstrating sequence specificity. However, some knockdown efficiency could still be measured in the presence of a single mismatch (e.g. shRNA-G8A on wt target). In the case of two mismatches, knockdown was dramatically reduced (shRNA-G8A on the G15A target) or even absent (shRNA-G15A on the G8A target). Most importantly, the shRNA-combi (wt+G8A+G15A) was indeed able to knockdown all three luciferase targets. These results are summarized in Table 1. We concluded that the 2^nd ^generation shRNAs are active inhibitors and that they act in a sequence-specific manner.

**Table 1:** Inhibition of luciferase expression

	shRNAs			
	
Target	wt	G8A	G15A	wt+G8A+G15A
**wt**	++^a^	+/-	+	++

**G8A**	+	++	-	++

**G15A**	+	-	++	++

^a ^score of RNAi activity

### HIV-1 inhibition studies with the 2^nd ^generation shRNAs

**Figure 3:**
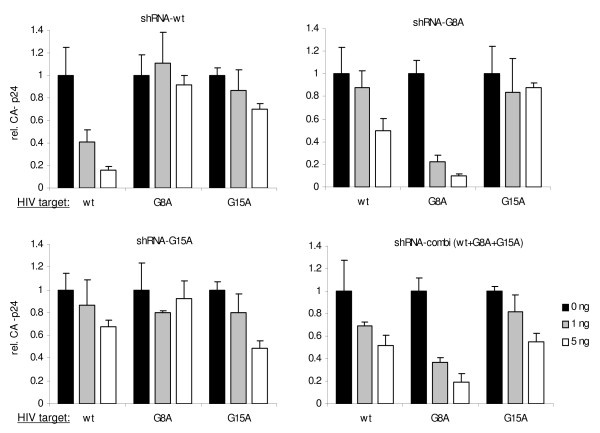
**Inhibition of HIV-1 production by 2^nd ^generation shRNA**. 293T cells were co-transfected with 100 ng pLAI, 0.5 ng of renilla luciferase plasmid and 0, 1 and 5 ng of the shRNA constructs. The CA-p24 level in culture supernatant was measured and renilla luciferase expression was measured to control for the transfection efficiency. Values are shown as percentage of the transfection without shRNA. Averages and standard deviations represent at least three independent transfections that were performed in quadruple.

We next tested whether the 2^nd ^generation shRNAs are capable to inhibit virus production of the escape variants. The G8A and G15A mutated HIV-1 molecular clones were generated by site-directed mutagenesis. Two amounts (1 and 5 ng) of the shRNA constructs were co-transfected with the wt and mutant HIV-1 molecular clones in 293T cells, and virus production was measured by CA-p24 ELISA in the culture supernatant at 48 hours post transfection (Fig. 3). A similar pattern was observed as in the luciferase reporter assay in Figure 2. Virus production was inhibited in a sequence-specific manner. Thus, the wt virus was affected by shRNA-wt, whereas the escape variants were inhibited by the respective 2^nd ^generation shRNA (G8A or G15A). The shRNA-combi (wt+G8A+G15A) was able to inhibit the production of all three viruses. The results are summarized in Table 2. The impact of a single mismatch in the RNAi duplex seems more dramatic in the virus production assay than the luciferase assay. Most importantly, the 2^nd ^generation shRNAs represent potent inhibitors against the perfectly matched target sequence.

**Table 2:** Inhibition of HIV-1 production

	shRNAs			
	
Target	wt	G8A	G15A	wt+G8A+G15A
**wt**	++^a^	-	-	++

**G8A**	+	++	-	++

**G15A**	+/-	-	+	+

**G8A+G15A**	-	-	-	-

^a ^score of RNAi activity

**Figure 4:**
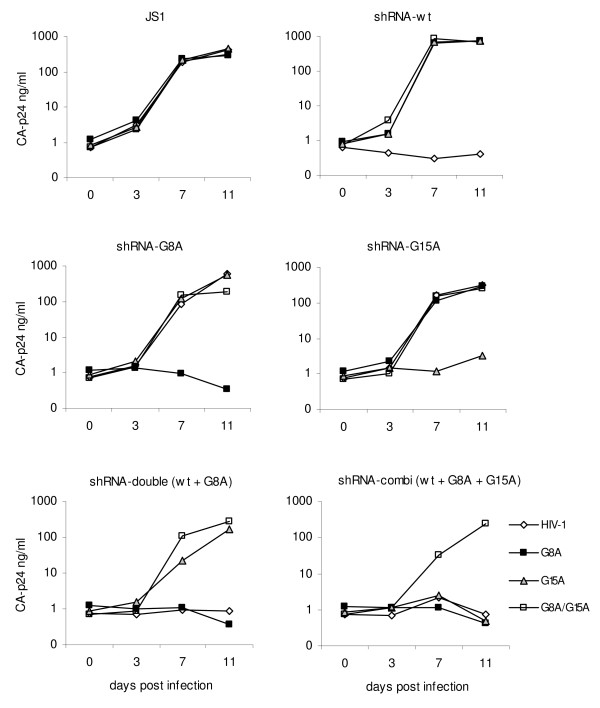
**Potent inhibition of HIV-1 replication by 2^nd ^generation shRNA**. Stable cell lines (SupT1) expressing the shRNA inhibiters (wt, G8A, G15A or combined) were infected with wt HIV-1 (1 ng CA-p24), the escape viruses (G8A and G15A) or the double mutant (G8A/G15A). Virus replication was monitored over time. SupT1 cells with the empty lentiviral vector JS1 served as positive control. Results were obtained in three independent infection experiments.

To perform HIV-1 replication assays, the SupT1 T cell line was transduced with the lentiviral vector to allow stable shRNA expression. A low multiplicity of infection (0.15) was used to ensure that cells obtain a single copy of the shRNA cassette. SupT1 cells transduced with the empty lentiviral vector (JS1) served as control. Next to the three single shRNA constructs and the shRNA combination, a shRNA-double (wt+G8A) was used as an additional control. Furthermore, a double mutant virus (G8A+G15A) was included. These different SupT1 cells were infected with the set of HIV-1 variants, and virus spread was monitored by CA-p24 production (Fig. 4). The wt and three mutant viruses (G8A, G15A, G8A+G15A) replicated efficiently and reached peak infection after 7 days. However, no replication of HIV-1 wt was observed in the SupT1-shRNA-wt cells, although all mutant viruses reached peak infection at day 7. Sequence-specific inhibition was also observed for the other cell lines. Thus, mutant virus replication was completely blocked by the corresponding 2^nd ^generation shRNA. On the shRNA-double (wt+G8A) cell line, the G15A and G8A/G15A mutant viruses were able to replicate efficiently, which makes sense as the G15A mutation causes a mismatch. On the shRNA-combi (wt+G8A+G15A) cells only the G8A/G15A mutant virus was able to replicate, as expected because the target sequence of the double mutant virus always contains at least one mismatch with the shRNA. These virus replication results are summarized in Table 3.

**Table 3:** Inhibition of HIV-1 replication

	shRNAs				
	
Target	wt	G8A	G15A	wt+G8A	wt+G8A+G15A
**wt**	++^a ^(0)^b^	- (1)	- (1)	++ (0.1)^c^	++ (0.1.1)^c^

**G8A**	- (1)	++ (0)	- (1)	++ (1.0)	++ (1.0.1)

**G15A**	- (1)	- (1)	++ (0)	- (0.1)	++ (1.1.0)

**+G8A+G15A**	- (2)	- (1)	++ (1)	- (2.1)	- (2.1.1)

^a ^score of RNAi activity

^b ^number of mismatches

^c ^number of mismatches per shRNA

### Blocking of popular HIV-1 escape routes by 2^nd ^generation shRNAs

**Figure 5:**
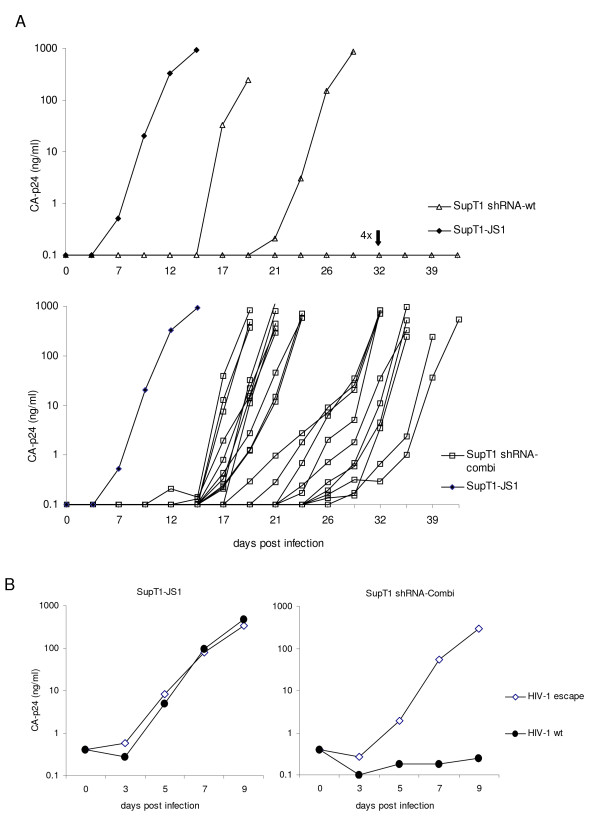
**HIV-1 escapes from the 2^nd ^generation combination shRNAs**. (A) Stable cell lines (SupT1) expressing the shRNA-wt or shRNA-combi (wt+G8A+G15A) were infected with wt HIV-1 (1 ng CA-p24). Virus replication was monitored over time. SupT1 cells with the empty lentiviral vector JS1 served as positive control. (B) Control SupT1 cells and cells expressing shRNA-combi (wt+G8A+G15A) were infected with the escape variant (1 ng CA-p24) and wt HIV-1.

The results obtained thus far support the 2^nd ^generation concept, but it remains to be tested whether virus evolution is indeed affected or blocked by this approach. We therefore challenged the SupT1-shRNA-combi cells (wt+G8A+G15a) with HIV-1. As controls, we infected SupT1-shRNA-wt cells that previously showed good inhibition but eventual viral escape, and SupT1-JS1 control cells without antiviral RNAi pressure. We infected 21 independent cultures of SupT1-shRNA-combi (wt+G8A+G15A), 6 SupT1-shRNA-wt cultures and 2 SupT1-JS1 cultures with an equal amount of HIV-1 (1 ng CA-p24). Virus replication was monitored by CA-p24 measurement in the culture supernatant and visual inspection for virus-induced syncytia (Fig. 5). Peak infection of the control SupT1--JS1 cells was reached within 10 days. Potent inhibition of virus replication was observed for all shRNA expressing cells for at least 14 days, but virus emerged in many cultures at a later time point. Viral replication was eventually observed in 2 of 6 SupT1-shRNA-wt cultures and all SupT1-shRNA-combi (wt+G8A+G15a) cultures. No virus replication was measured in the remaining SupT1 shRNA-wt cultures up to 42 days post infection, when the experiment was stopped. These results may seem surprising as the single shRNA therapy seems to do much better than the combination approach. However, one should note that our shRNA-combination was designed to restrict virus evolution, and not designed to achieve maximal virus inhibition. In fact, one could argue that the 2^nd ^generation shRNAs, which have a mismatch with the HIV-1 RNA genome, will dilute the potent inhibition of the primary shRNA.

Viral breakthrough replication may indicate the selection of escape variants that are resistant to the shRNA inhibitor. To confirm whether the emerging viruses have a resistant phenotype, fresh SupT1 shRNA and control cells were infected with cell free virus collected at the peak of infection. One example is shown in Figure 5B. On the control cells, wt virus (HIV-1 wt) and escape virus (HIV-1 escape) replicated equally well, whereas on the restricted SupT1-shRNA-combi (wt+G8A+G15A) cells only the escape virus replicated efficiently, confirming a resistant phenotype of the selected virus. A similar resistant phenotype was measured for all 21 cultures. Thus, plenty of candidate escape viruses were selected to test if the 2^nd ^generation approach was able to block certain escape routes.

**Figure 6:**
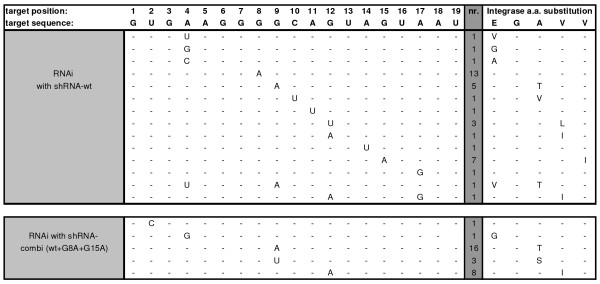
**Escape mutations in the 19-nt HIV-1 integrase target region**. The 19 nt target is shown. Mutations were scored in multiple evolution cultures. The frequency of each escape mutation is listed in the middle column (marked gray). Amino acid changes in the integrase enzyme are shown in the right column. The upper panel shows the escape profile on the target sequence induced by shRNA-wt (21 cultures from [17] and 2 from this study). The lower panel shows the more restricted escape profile for shRNA-combi (wt+G8A+G15A) observed in 21 cultures.

A large-scale sequence analysis was performed to examine the viral escape strategies. The 19-nt target sequence of the integrase gene and the flanking regions were sequenced for all 21 evolved HIV-1 variants. HIV-1 proviral sequences were PCR amplified from infected cells and cloned. At least 8 clones per culture were sequenced, yielding numerous candidate escape sequences. True escape mutations will become dominant in the viral quasispecies and should thus be present in multiple clonal sequences per culture. Therefore, only sequences that occurred in at least two clonal sequences per culture were scored. This rule was also applied when more than one type of mutant was present in a single culture (mixed culture). The evolution studies with shRNA-wt revealed G8A and G15A as favorite escape routes (Fig. 6, upper panel). The presence of the 2^nd ^generation shRNAs effectively blocked these G8A and G15A routes, which are not observed anymore (Fig. 6, bottom panel). Viral escape did nevertheless occur, apparently by alternative routes. Under pressure of the 2^nd ^generation shRNAs, the most frequent mutations are G9A (observed 16×) and G12A (8×). In fact, these routes were already observed in the shRNA-wt experiment as minority escape routes (Figure 6, upper panel). By comparing the two panels in Figure 6, it is also clear that a reduced number of escape routes allow HIV-1 to escape from shRNA-combi versus the single shRNA-wt inhibitor. Three new minority escape routes were observed: G9U (3×), A4G (1×) and T2C (1×). Changes in the amino acids of the integrase enzyme due to these escape mutations are depicted in the right column of Figure 6. No escape mutations were observed outside the target region. Other characteristics of this evolution experiment confirm previous findings, including the preference for G-to-A mutations as driver of HIV-1 escape [19].

**Figure 7:**
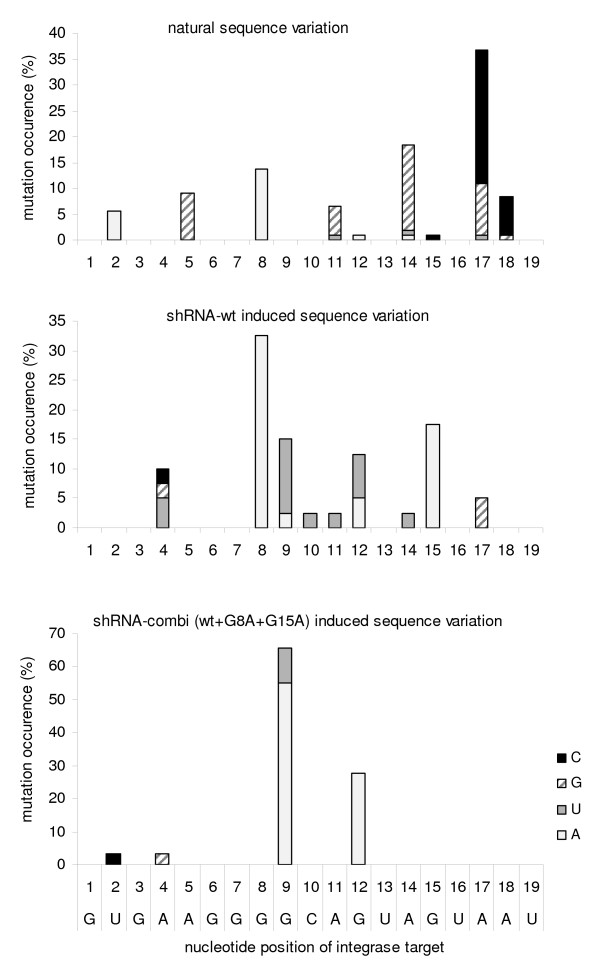
**Sequence variation in the integrase target region**. The natural variation of HIV-1 in the target region (upper panel) is compared with the sequence variation under RNAi pressure by a single shRNA-wt (middle panel) or the shRNA-combi (wt+G8A+G12A) (lower panel). The natural sequence variation was derived from the Los Alamos HIV-1 database. Each bar represents the frequency that the mutation occurs at the indicated position.

These results indicate that the shRNA-combi (wt+G8A+G15A) regimen can effectively block viral escape routes, such that HIV-1 is forced to use alternative escape strategies. We plotted the results as relative values for the occurrence of the specific mutation within the integrase target sequence (Fig. 7). The results show the imposed restriction of the viral escape possibilities by the 2^nd ^generation approach (bottom panel) in comparison with the original single shRNA therapy (middle panel). The natural sequence variation in this integrase encoding sequence is also plotted (top panel).

## Discussion

When the HIV-1 RNA genome is attacked by potent therapeutic shRNAs, the virus escapes by selecting a point mutation within the target sequence [7,8]. A combination approach with multiple shRNA inhibitors can be developed to prevent viral escape [11]. In this study, we tested a different strategy, which can be employed when it is known that the virus can only use a limited number of escape routes. In this scenario, one can propose a combinatorial RNAi approach that targets both the wt sequence and the most favorite escape mutants, thus blocking viral escape. We tested this concept for a potent shRNA that attacks a well conserved sequence encoding the HIV-1 integrase enzyme, and for which only two major escape routes were described in massive evolution studies [17]. We now designed the two matching shRNA variants, which we called 2^nd ^generation shRNAs, that anticipate viral escape. We show that the 2^nd ^generation shRNAs are efficient inhibitors that give sequence-specific knockdown of the target. When 2^nd ^generation shRNAs and the primary shRNA-wt are combined they potently inhibit viral replication and effectively block the two favorite escape routes. However, viral evolution is redirected towards the emergence of novel escape mutants. These secondary escape routes were already seen as minority routes in the original evolution study, but their prevalence is increased when the major routes are blocked.

We compared the RNAi-induced sequence variation with that of natural HIV-1 strains (Fig. 7). The shRNA-wt induced sequence variation (G8A and G15A) does in fact resemble the sequence variation in natural HIV-1 strains. We previously argued that the same mutations emerge in these two different evolution settings because these changes do not affect the integrase enzyme function and the viral replicative capacity [17]. Indeed, the most prominent G8A variation causes a silent codon change and will not affect the integrase enzyme (Fig. 6). In contrast, the second best escape route (G15A) and the newly observed escape routes upon 2^nd ^generation pressure (G19A and G12A) are non-silent and the amino acid substitutions in the integrase protein may negatively affect viral fitness. As indicated earlier, the integrase target sequence is highly conserved among virus isolates. Inspection of 178 viral isolates (including multiple subtypes) in the 2009 HIV-1 sequence compendium indicates only 2 isolates with a single amino acid substitution: 248V changes to 248I (isolate cxp.GR.x.GR17) and 249V changes to 249L (isolate A1.SE.95.SE8891) [20]. The amino acid substitutions selected in the 245-EGAVV-249 motif (Fig. 6, right column) have not been studied earlier in mutagenesis studies. Resistance mutations to the integrase inhibitors Raltegravir and Elvitegravir do not map to these residues [21,22]. Thus, it would be of interest to test whether the 2^nd ^generation therapy selects for sub-optimal HIV-1 variants with reduced replication fitness and potentially reduced pathogenicity.

In theory, additional 2^nd ^generation shRNAs could be designed against these new escape routes to prevent viral escape. This would necessitate the design of a combinatorial RNAi attack with at least 5 shRNAs (wt + 4 × 2^nd ^generation shRNAs). On the other hand, it seems very difficult to contain virus evolution as we still observed other minority escape routes, even though we target one of the most conserved viral sequences. It has been described that HIV-1 can also escape from RNAi pressure by mutations outside the target sequence that trigger an alternative structure in the RNA genome that restricts RNAi attack [16]. This escape route may be rather exotic because it depends on the ability of the RNA sequences to adopt a restrictive RNA structure, but it does indicate that mutational escape is not necessarily restricted to the 19-nucleotide target sequence.

A disadvantage of the 2^nd ^generation approach is that it has a negative effect on the initial level of virus inhibition. Our experiments indicate that the G8A and G15A shRNA inhibitors inhibit the wt virus only partially. Saturation of the RNAi machinery, in particular the RISC complex, with these sub-optimal inhibitors will dilute the effect of the potent wt inhibitor. There will be competition between the shRNAs for the available RISC complexes. This explains why viral escape was delayed with the single potent shRNA-wt compared to the shRNA-combi (wt+G8A+G15A). These combined arguments stress the practical limitations of the 2^nd ^generation RNAi approach. The use of multiple shRNAs against different viral targets therefore seems a better combinatorial strategy against HIV-1 [11,23,24]. In such a therapeutic scenario, all shRNAs will be potent viral inhibitors and viral escape is prevented because it is too difficult for the virus to acquire mutations in all targets at the same time.

The 2^nd ^generation principle could perhaps be combined with other therapeutic strategies, including regular antiretroviral drugs, to skew viral evolution. For most of the antiretroviral drugs the HIV-1 escape mutations are known [21,22]. For instance, only two escape mutations have been reported for the RT inhibitor 3TC, which could be targeted and thus prevented by 2^nd ^generation RNAi. This approach has been successfully used to inhibit hepatitis B virus replication in vitro [25]. As seen in this study, the virus may still escape through alternative escape routes, but these HIV-1 variants may exhibit reduced drug-resistance and/or reduced replication capacity, which may provide clinical benefit.

This study provides additional insight on the level of sequence complementarity between the siRNA and HIV-1 target that is required for an effective RNAi attack [26-29]. The data presented in this and our previous studies [16,17] show that a single mismatch will allow HIV-1 to replicate under shRNA pressure. Tables 1, 2 and 3 summarize the RNAi inhibitory effect measured in the different assays systems. In relatively simple transient assays with a luciferase reporter, nucleotide mismatches do only partially affect the RNAi activity of a shRNA (Table 1). The more complex transient assay of virus production yields an intermediate effect of mismatches (Table 2). The biggest impact of a mismatch was scored in HIV-1 replication (Table 3). The effects are likely to be enhanced in the viral context because virus replication is a multi-cycle assay. This means that HIV-1 is an extremely sensitive RNAi target and single mutations can frustrate the RNAi attack. Modifications of the shRNA reagent, e.g. construction of miRNA-like inhibitors, may induce such mutation-tolerance [30-32]. There may also be an effect of the viral Tat protein as an RNAi suppressor [9,33,34].

The 2^nd ^generation RNAi approach was successful in blocking particular HIV-1 escape mutations and shows promise as a new antiviral option in the battle against HIV-1 and its ability to acquire drug resistance mutations. We were able to steer virus evolution towards escape mutations that may be less favorable for the virus in terms of replication fitness or the level of shRNA-resistance. The 2^nd ^generation approach may thus lead to the selection of attenuated HIV-1 variants, resulting in a lower viral load and delayed disease progression.

## Conclusion

The 2^nd ^generation shRNA strategy anticipates HIV-1 escape by designing secondary shRNAs that form a complete match with the most popular viral escape sequences. We indeed demonstrated that two dominant escape routes were effectively blocked in prolonged viral challenge experiments. However, HIV-1 escape did still occur, and we observed the upgrading of two previous minority escape paths into major escape routes. Consequently, HIV-1 evolution was effectively skewed by the designer RNAi reagents. These results highlight different aspects of HIV-1 evolution and provide insight to develop a durable RNAi- based therapy.

## Methods

### Plasmid construction

The lentiviral vector JS1 (pRRLcpptpgkgfppreSsin) and the construction of single shRNA (wt, G8A, G15A), double shRNA (wt+G8A) and triple shRNA (wt+G8A+G15A) derivatives were described previously [11,35,36]. The integrase shRNA was previously named shRNA-pol47, but this was changed to shRNA-wt in the context of this study. The shRNA-wt expression plasmid targets the wt HIV-1 sequence and is based on pSUPER (OligoEngine, Seattle, WA) with the human H1 polymerase III promoter. The shRNA-G8A variant targets the G8A escape virus and is based on psiRNA-h7Skhygro G1 (Invivogen, San Diego, CA) with the human 7SK polymerase III promoter. The shRNA-G15A variant targets the G15A escape virus and is based on pSilencer 2.0-U6 (Ambion, Austin, TX) with the human U6 polymerase III promoter. The shRNA expression plasmids were constructed by inserting annealed oligonucleotides into the appropriate restriction sites. Additional restriction sites were inserted 3' of the transcription termination sites of the U6 and 7SK constructs to facilitate combinatorial cloning of the shRNA constructs (BglII, ZraI, ClaI, XhoI for U6 and SalI, XhoI for 7SK). The shRNA cassettes were excised with SmaI/XhoI and inserted in the multiple cloning site (EcoRV/XhoI) of JS1 to create JS1-shRNA.

The firefly luciferase (Luc) reporter plasmids, containing HIV-1 target sequences of wt or mutants G8A and G15A, were constructed by insertion of a 50- to 70-nucleotide HIV-1 fragment, with the 19- nucleotide target sequence in the centre, in the EcoRI and PstI sites of pGL3.

The full-length HIV-1 molecular clone pLAI [37] was used to produce wt virus and to study its inhibition by the antiviral shRNAs. The G8A and G15A mutant HIV-1 LAI molecular clones were generated by site-directed mutagenesis (24). pLAI was digested with EcoRI, and the integrase fragment (position 4732 to 5827) was cloned into pBSK to generate pBSK-in. Mutations were introduced into pBSK-in by site-directed mutagenesis and verified by sequence analysis, and the mutant fragment was subsequently cloned back into pLAI.

### Cell culture

Human embryonic kidney 293T adherent cells were grown as monolayer in Dulbecco's modified Eagle's medium (Invitrogen, Carlsbad, CA) supplemented with 10% fetal calf serum, penicillin (100 U/ml) and streptomycin (100 μg/ml) in a humidified chamber at 37°C and 5% CO2. SupT1 suspension T cells were grown in Advanded Rosewell Park Memorial Institute medium (Invitrogen, Carlsbad, CA) supplemented with l-glutamine, 1% fetal calf serum, penicillin (30 U/ml) and streptomycin (30 μg/ml), in a humidified chamber at 37°C and 5% CO_2_.

### Transfection experiments

Co-transfections of pLAI or pGL-3 (Firefly luciferase reporter) and the shRNA vector were performed in a 96-well format. Per well, 2 × 10^4 ^293T cells were seeded in 100 μl DMEM with 10% FCS without antibiotics. The next day, 100 ng of pLAI (or 25 ng of pGL-3), 0-5 ng of shRNA vector, and 0.5 ng of pRL (Renilla luciferase) were transfected with 0.5 μl Lipofectamine 2000 in a reaction volume of 50 μl according to the manufacturer's instructions (Invitrogen).

Two days after pLAI transfection the supernatant was harvested, virus was inactivated and CA-p24 ELISA was performed. The cells were lysed for Renilla luciferase activity measurements with the Renilla Luciferase Assay System (Promega). To correct for transfection variation, the CA-p24 values were divided by the Renilla values. We set the condition that for an experiment to be valid the ratio between the highest and the lowest Renilla values should differ by less than a factor of 2.

Two days after pGL-3 transfection, cells were lysed to measure firefly and Renilla luciferase activities with the Dual-Luciferase Reporter Assay System (Promega, Madison, WI) according to the manufacturer's instructions.

### Lentiviral vector production and transduction

The lentiviral vector was produced as previously described [11]. Briefly, the vector was made by co-transfection of lentiviral vector plasmid and packaging plasmids pSYNGP, pRSV-rev, and pVSV-g with Lipofectamine 2000 (Invitrogen, Carlsbad, CA). After transfection, the medium was replaced with OptiMEM (Invitrogen, Carlsbad, CA). The lentiviral vector containing supernatant was collected after two days and aliquots were stored at -80°C. Next, SupT1 cells were transduced with a multiplicity of infection (MOI) of 0.15. Two to three days after transduction, live cells were sorted with FACS and GFP-positive cells were selected.

### HIV-1 infection and HIV-1 evolution experiments

HIV-1 LAI and the shRNA-wt resistant virus variants G8A and G15A were produced by transfection of the molecular clones in 293T cells. Virus production was measured by CA-p24 enzyme-linked immunosorbent assay. SupT1 cells (5 ml cultures, 2.5 × 10^6 ^cells or 24 wells plate, 2 × 10^5 ^cells in 1 ml) were infected with the HIV-1 isolate LAI or G8A/G15A escape variants, the viral input ranged from 0.1 - 1 ng CA-p24. Virus spread was monitored by syncytia formation followed by measuring CA-p24 production.

When virus replication was observed in the HIV-1 evolution experiments, cell-free virus was passaged to uninfected control and SupT1-shRNA cells and virus replication was monitored. At peak infection, cell and supernatant samples were stored at -80°C or directly used for sequencing analysis of the proviral target regions. Cellular DNA of the infected cells with the integrated provirus was isolated as previously described [38]. Integrated proviral DNA sequences were PCR amplified with the primer pairs IN sense (GAAGCAGAAGTTATCCCAGCAGAGACAGGGC; position 4567) and antisense (CCCAAGCTTCTAATCCTCATCCTGTCTACTTGCC; position 5157). The PCR products were gel purified and cloned into the pCR2.1 TOPO vector and subsequently sequenced with the T7 or M13R primers.

## Competing interests

The authors declare that they have no competing interests.

## Authors' contributions

All authors participated in the design of the study, NCTS performed the experiments, NCTS and BB drafted the manuscript.
